# Investigation of Elimination Rate, Persistent Subpopulation Removal, and Relapse Rates of Mycobacterium tuberculosis by Using Combinations of First-Line Drugs in a Modified Cornell Mouse Model

**DOI:** 10.1128/AAC.02548-15

**Published:** 2016-07-22

**Authors:** Yanmin Hu, Henry Pertinez, Fatima Ortega-Muro, Laura Alameda-Martin, Yingjun Liu, Alessandro Schipani, Geraint Davies, Anthony Coates

**Affiliations:** a Institute for Infection and Immunity, St. George's, University of London, London, United Kingdom; b Department of Molecular and Clinical Pharmacology, University of Liverpool, Liverpool, United Kingdom; c GlaxoSmithKline Research and Development, Diseases of Developing World, Tres Cantos, Madrid, Spain

## Abstract

Currently, the most effective tuberculosis control method involves case finding and 6 months of chemotherapy. There is a need to improve our understanding about drug interactions, combination activities, and the ability to remove persistent bacteria using the current regimens, particularly in relation to relapse. We aimed to investigate the therapeutic effects of three main components, rifampin (RMP), isoniazid (INH), and pyrazinamide (PZA), in current drug regimens using a modified version of the Cornell mouse model. We evaluated the posttreatment levels of persistent Mycobacterium tuberculosis in the organs of mice using culture filtrate derived from M. tuberculosis strain H37Rv. When RMP was combined with INH, PZA, or INH-PZA, significant additive activities were observed compared to each of the single-drug treatments. However, the combination of INH and PZA showed a less significant additive effect than either of the drugs used on their own. Apparent culture negativity of mouse organs was achieved at 14 weeks of treatment with RMP-INH, RMP-PZA, and RMP-INH-PZA, but not with INH-PZA, when conventional tests, namely, culture on solid agar and in liquid broth, indicated that the organs were negative for bacteria. The relapse rates for RMP-containing regimens were not significantly different from a 100% relapse rate at the numbers of mice examined in this study. In parallel, we examined the organs for the presence of culture filtrate-dependent persistent bacilli after 14 weeks of treatment. Culture filtrate treatment of the organs revealed persistent M. tuberculosis. Modeling of mycobacterial elimination rates and evaluation of culture filtrate-dependent organisms showed promise as surrogate methods for efficient factorial evaluation of drug combinations in tuberculosis in mouse models and should be further evaluated against relapse. The presence of culture filtrate-dependent persistent M. tuberculosis is the likely cause of disease relapse in this modified Cornell mouse model.

## INTRODUCTION

Tuberculosis (TB) remains a major killer worldwide and is responsible for approximately two million deaths annually (1). The main obstacle for successful disease control resides in the ability of M. tuberculosis to persist in the host despite host immune responses and chemotherapy. Prolonged multidrug antimicrobial therapy is necessary to achieve a cure, which leads to poor patient compliance, high relapse rates (7 to 13%), and the emergence of drug resistance (2). Although short-course TB therapy has been in clinical use for nearly 4 decades, the drug interactions and the ability to remove persistent bacteria with the current regimens have not been clearly demonstrated. Previous work in the murine Cornell model has shown that after 7 weeks of intensive treatment with isoniazid (INH) and pyrazinamide (PZA) to induce a latent infection, the follow-up treatment with rifampin (RMP) alone, RMP-INH, RMP-PZA, or RMP-INH-PZA exhibited very similar antituberculosis activities (3). However, another study found that when mice were treated with INH-RMP-PZA, INH-RMP, or RMP-PZA for 6 months, the RMP-PZA-treated group demonstrated significantly lower relapse rates than the INH-RMP-PZA or INH-RMP group (4). This study suggested that INH antagonized the actions of RMP-PZA (4) because INH in the regimen significantly reduced the *C*_max_ (maximum concentration of drug in serum) and the AUC (area under the serum concentration-time curve) of RMP in the mice (4), leading to higher relapse rates. The antagonism between INH and RMP-PZA was due to a negative interaction between INH and PZA in the combination, and the effect was INH dose dependent (5). It was not clear what interaction INH has with each of the components in the regimens. To provide greater clarity, it is important to identify and evaluate the level of persistent bacilli after chemotherapy. This information is of clinical importance, since combination therapy involving RMP-INH-PZA is commonly employed. Using appropriate drug combinations has the potential to maximize therapeutic effects while minimizing side effects of multiple-drug therapy. Furthermore, evaluation of posttreatment persister levels may serve as a biomarker to predict relapse rates (6). In this study, we examined the therapeutic effects of each of the components singly and in two-drug and three-drug combinations using a modified Cornell mouse model. We evaluated persistent M. tuberculosis using culture filtrate, which was shown by others (7) to contain resuscitation-promoting factors (RPF) in mouse organs, from a population of mice from a sample of apparently culture-negative organs after long-term chemotherapy.

## MATERIALS AND METHODS

### Bacterium and growth conditions.

M. tuberculosis strain H37Rv was mouse passaged and grown in 7H9 medium supplemented with 10% albumin dextrose complex (ADC; Becton and Dickinson, United Kingdom) containing 0.05% Tween 80 at 37°C without disturbance for 15 days. The culture was subsequently frozen at −70°C for storage. To determine the viable counts prior to infection, CFU counting was performed prior to freezing and once again after thawing. CFU counts were carried out by plating serial 10-fold dilutions of the cultures on 7H11 agar medium supplemented with oleic albumin dextrose complex (OADC; Becton and Dickinson, United Kingdom). Colonies were counted after incubation of the plates at 37°C for 3 to 4 weeks, and viability was expressed as log CFU/ml. The cultures were subsequently diluted in phosphate-buffered saline (PBS) and used for inoculations in mice.

### Modified Cornell mouse model.

Rifampin, isoniazid, and pyrazinamide were tested singly or in double (RMP-INH, RMP-PZA, and INH-PZA) or triple (RMP-INH-PZA) combinations using a modified Cornell mouse model, which was based on the model previously established at Cornell University (8, 9). The model was conducted using the experimental design and procedure described below.

### (i) Infection of mice.

Female BALB/c mice (6 to 8 weeks old) were obtained from Harlan United Kingdom Ltd. A total of 364 mice were infected intravenously via the tail vein with 1.2 × 10^5^ CFU of mouse-passaged M. tuberculosis strain H37Rv per mouse as described previously (8, 10, 11). The animal husbandry guidelines and all animal experiments were performed according to the Animals Scientific Procedures Act, 1986 (an Act of the Parliament of the United Kingdom 1986 c. 14) (Home Office Project license Number 70/7077) with approval from the St. George's, University of London, ethics committee.

### (ii) Chemotherapy.

As shown in Table 1, mice were randomly allocated into eight groups. The control group consisted of infected and untreated mice; 4 of these were sacrificed 2 h after infection (D0), and then 4 were killed at the beginning of treatment and at 14 and 21 days after infection (D14 and D21, respectively). The treatment groups were single-drug treatment groups, each containing 16 mice receiving RMP, INH, or PZA, respectively, for 8 weeks, and combination groups, each containing 76 mice that were administered RMP-INH, RMP-PZA, INH-PZA, or RMP-INH-PZA for 14 weeks. Single-drug therapy started 14 days after infection, when a large bacterial load in the organs (the mean CFU counts reached 10^7^ per lung or spleen) had been achieved with visible symptoms of disease. Combination therapy started at 21 days after infection. All groups were treated by daily oral administration (0.2 ml) for 5 days per week at dosages of 10 mg/kg of body weight RMP, 25 mg/kg INH, or 150 mg/kg PZA. The drug suspensions were prepared freshly for the daily dose. In the combination containing RMP, RMP was administered 1 h before the other drugs to avoid drug-drug interactions (4). Immediately after termination of 14 weeks of chemotherapy, the remaining mice were administered 0.5 mg/mouse of hydrocortisone acetate by daily oral administration for 8 weeks to suppress host immune response. The counting of CFU from lungs and spleens was performed to determine disease relapse.

**TABLE 1:** Mouse tuberculosis experimental design

Treatment group^*a*^	Total no. of mice^*b*^	D0	D14	D21	W2	W4	W6	W8	W11	W14	W22^*c*^
Control	12	4	4	4							
RMP	16				4	4	4	4			
INH	16				4	4	4	4			
PZA	16				4	4	4	4			
RMP-INH	76				8	8	8	8	10	10	24
RMP-PZA	76				8	8	8	8	10	10	24
INH-PZA	76				8	8	8	8	10	10	24
RMP-INH-PZA	76				8	8	8	8	10	10	24

a Mice were intravenously infected on day 0 (D0). Treatment commenced 14 days after infection for single-drug therapy (D14) and 21 days after infection for combination therapy (D21). Dosages for each drug were the following: RMP, 10 mg/kg; INH, 25 mg/kg; and PZA, 150 mg/kg.

b Total mice includes mice that were infected and treated and excludes mice that died of natural causes during the course of treatment.

c Eight weeks of hydrocortisone treatment after 14 weeks of treatment.

### (iii) Assessment of infection and treatment efficacy.

As seen in Table 1, to examine M. tuberculosis infection and baseline CFU counts before initiation of chemotherapy, 4 untreated control mice were sacrificed at 2 h, 14 days, and 21 days after infection, respectively. For the assessment of treatment efficacy, 4 mice were sacrificed at 2, 4, 6, and 8 weeks posttreatment for single-drug treatment to monitor CFU counts. For combination therapy, a sample of 8 mice was sacrificed at 2, 4, 6, and 8 weeks, and 10 mice were sacrificed at 11 and 14 weeks of treatment (Table 1). Lungs and spleens from mice were removed rapidly after sacrifice and a sterile autopsy was performed. The organs were transferred into 2-ml tubes, each containing 1 ml sterile distilled water and 2-mm-diameter glass beads. Lungs and spleens of mice were homogenized using a reciprocal shaker (Thermo Hybaid Ltd.) for 40 s at a speed of 6.5. CFU counts from each lung and spleen sample were performed using serial dilutions of the homogenates. At the 14th week of treatment, the entire-organ homogenates (the total volume of each organ homogenate was approximately 1.5 ml, including the organ and 1 ml of water) from the 10 mice were aliquoted equally into three tubes. Tube 1 was used for CFU counting by addition of the homogenate to 2 ml of sterile distilled water, followed by plating out the entire-organ homogenate suspension on 6 selective 7H11 agar plates. Tube 2 was used for culturing in 5 ml of selective Kirchner liquid medium by the addition of 200 U/ml polymyxin B, 100 mg/liter carbenicillin, 20 mg/liter trimethoprim, and 10 mg/liter amphotericin B (Selectatab; Mast Diagnostica GmbH) for 4 weeks with subsequent subculturing of the entire culture onto Löwenstein-Jensen slopes for a further 4 weeks. Tube 3 was used for resuscitation of persistent bacteria. Culture-negative organs were defined as no colonies grown on 7H11 agar plates and no growth in selective Kirchner liquid medium following inoculation on Löwenstein-Jensen slopes.

### Selection of RMP- and INH-resistant mutants in mice.

At the 4th, 6th, and 8th week posttreatment, mouse lung and spleen homogenates were plated on 7H11 plates containing RMP or INH concentrated at 2-fold serial dilutions from 1 to 64 mg/liter. Colonies from the plates containing MICs higher than 4-fold were picked and regrown in 7H9 medium. MIC was retested on RMP or INH containing 7H11 agar plates.

### Resuscitation of M. tuberculosis in mouse lungs and spleens.

For resuscitation of M. tuberculosis grown in mouse organs, culture filtrates containing RPF were used as described previously (6, 7). M. tuberculosis H37Rv was grown in 7H9 medium for 15 to 20 days until an optical density of 1 to 1.5 was reached. The cultures were harvested by centrifugation at 3,000 × *g* for 15 min and sterilized by filtration with a 0.2-μm filter (Sartorius) twice. The sterilized culture filtrates were made selective by addition of 200,000 U/liter polymyxin B, 100 mg/liter carbenicillin, 20 mg/liter trimethoprim, and 10 mg/liter amphotericin B (Selectatab; Mast Diagnostica GmbH) and immediately used for broth counting of the most probable number (MPN) of the bacilli (7). Broth counting of lungs and spleens after 14 weeks of combination therapy was performed with serial 10-fold dilutions in triplicate in which 0.5 ml of tissue homogenates was added to 4.5 ml of the culture filtrates. At 10-day intervals over a 2-month period of incubation at 37°C, the broth cultures were examined for visible turbidity changes. Growth of M. tuberculosis in turbid tubes was confirmed by colonial morphology on 7H11 agar plates. The MPN of viable bacilli was then estimated from patterns of positive and negative tubes (7). The absence of microorganisms other than mycobacteria from turbid tubes was confirmed by plating on blood agar medium (Oxoid) and Sabouraud dextrose agar (Oxoid). In order to assess the sterility of culture filtrates free of M. tuberculosis, tubes containing culture filtrates were incubated at 37°C for 2 months to ensure the absence of M. tuberculosis from the culture filtrates.

### Statistical analysis.

**FIG 1:**
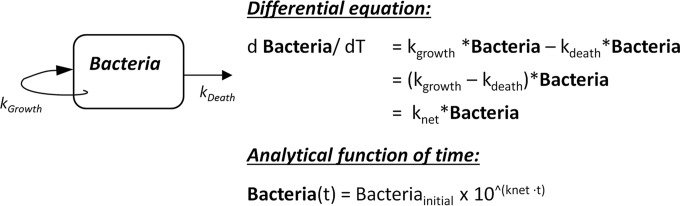
Simple mathematical model for exponential growth and decline of bacteria.

A simple model for mono-exponential bacterial growth and elimination (12) (Fig. 1) was fitted to the profiles of CFU versus time obtained experimentally. As simultaneously occurring exponential replication and death rates cannot be differentiated with this type of data, an exponential rate constant, *k*_net_, was estimated separately before treatment began (*k*_net_no_drug_, which would be a net positive value) and during treatment (*k*_net_with_drug_, which would be a negative value). During therapy, *k*_net_ is a first-order elimination rate constant which can be interpreted as the slope of the modeled line fit through the logarithmic transformation of the data (with units of these data being per week). Parameter estimation was carried out with nonlinear regression using the nonlinear least-squares optimization function “lsqnonlin” as part of the “pracma” package in the R statistical software language, with an objective function weighted by 1/(predicted value)^2^. Standard errors of parameter estimates were calculated using the method outlined by Landaw and DiStefano (13), with the Jacobian of model parameter sensitivities estimated using a numerical central difference method. The data sets, comprised from multiple individual subject animals, were treated as a naive pool for data analysis purposes (14) rather than using the average of the data at each time point. The significance of differences between model parameter estimates under different therapies was examined with pairwise Z-tests incorporating a Bonferroni correction of 21, where *P* values of <0.002 would be considered significant. The significance of differences between the relapse rates was determined with pairwise Fisher's exact tests with a Bonferroni correction of 6, with *P* values of <0.008 considered significant.

## RESULTS

### Survival of mice.

During treatment, 4 mice in the RMP-INH group died (1 at 9 weeks, 1 at 10 weeks, and 2 at 12 weeks), 2 mice in the RMP-PZA group died (1 at 10 weeks and 1 at 12 weeks), and 3 mice died in the INH-PZA group died (1 at 9 weeks, 1 at 10 weeks, and 1 at 13 weeks). The reason for the death was unknown but was most likely due to natural causes, such as tumor development or neurological disorders, and was unrelated to tuberculosis or treatment. As the time of death was uncertain and did not occur at the sampling time point, organ bacterial counts were not determined from these animals. No mortality was observed during the course of single-drug and RMP-INH-PZA treatments.

### Treatment with RMP, INH, and PZA singly and in two-drug or three-drug combination in a modified Cornell mouse model.

**FIG 2:**
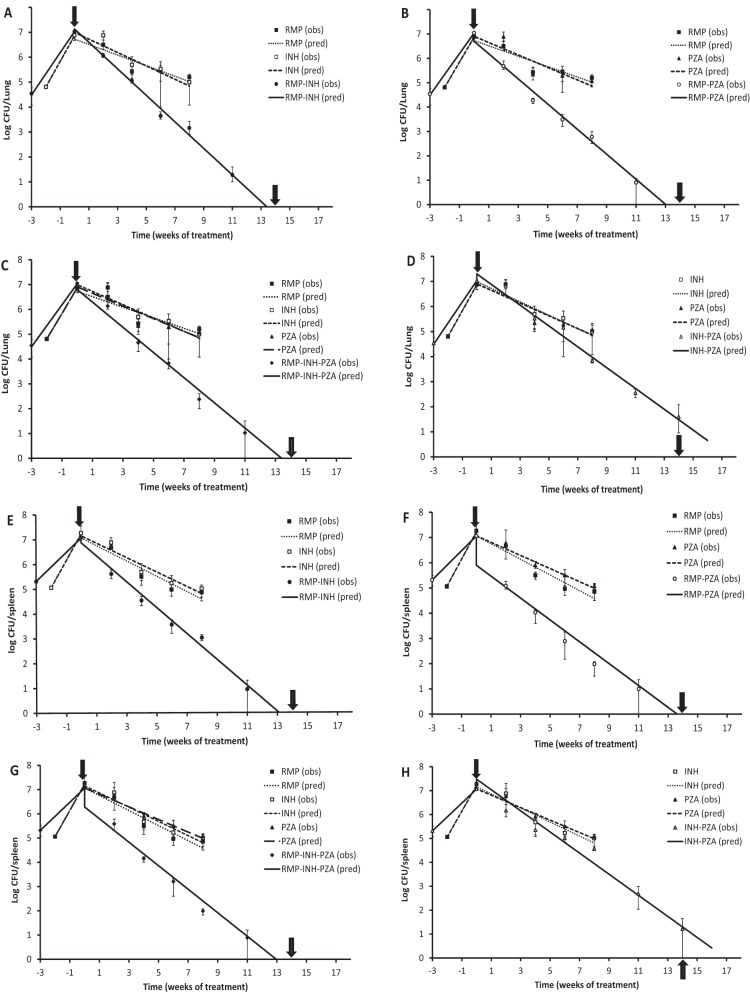
Treatment profiles of M. tuberculosis H37Rv with RMP, INH, and PZA singly or in combination in the modified Cornell mouse model. The results of a single experiment are shown with viability expressed as log CFU counts per lung or per spleen. Mice were infected intravenously at week −2 or −3, and the infection was allowed to progress for 2 or 3 weeks prior to treatment with RMP, INH, and PZA singly or in combination, indicated by solid black arrows, for 14 weeks (weeks 0 to 14). At weeks 2, 4, 6, 8, 11, and 14 posttreatment, CFU counts in the organs from each treatment group were estimated. Steroid treatment was started immediately after the termination of 14 weeks of antibiotic treatment, as indicated by arrows containing white lines. obs, observed; pred, predicted. (A) Treatment with RMP, INH, and RMP-INH in lungs. (B) Treatment with RMP, PZA, and RMP-PZA in lungs. (C) Treatment with RMP, INH, PZA, and RMP-INH-PZA in lungs. (D) Treatment with INH, PZA, and INH-PZA in lungs. (E) Treatment with RMP, INH, and RMP-INH in spleens. (F) Treatment with RMP, PZA, and RMP-PZA in spleens. (G) Treatment with RMP, INH, PZA, and RMP-INH-PZA in spleens. (H) Treatment with INH, PZA, and INH-PZA in spleens.

We investigated the effect of RMP, INH, and PZA singly and in double and triple combinations on the rates of bacterial eradication and relapse in a modified Cornell mouse model. The single dose of the drugs was tested in the animals for 8 weeks and terminated before resistant strain emergence (15). As shown in Tables 2 and 3 and Fig. 2, RMP at 10 mg/kg, INH at 25 mg/kg, and PZA at 150 mg/kg exhibited modest rates of bacterial eradication in both lungs and spleens, showing 99% kill (2-log reduction) at around 8 weeks. The exponential rate constants (logarithmic base 10) for net bacterial elimination during treatment (*k*_net_with_drug_) for RMP, INH, and PZA were −0.21, −0.27, and −0.26 for lungs and −0.31, −0.29, and −0.26 for spleens (Table 4), respectively. Notably, the drop in CFU counts in both lungs and spleens during the first 2 weeks of treatment with the singly dosed drugs was minimal, although over the complete time course of therapy a clear mono-exponential decline in CFU counts was observed. No RMP- or INH-resistant strains were isolated from 4 to 8 weeks of treatment. In addition, there was no significant difference in activities among the single-drug treatments (see Tables S1 and S2 in the supplemental material). Interestingly, treatment with RMP combined with INH (Fig. 2A and E) or PZA (Fig. 2B and F) accelerated the rate of bacterial eradication, showing 99% kill (Table 2 and 3) at 4 weeks of treatment for RMP-INH and at about 3 weeks for RMP-PZA, with the estimation of *k*_net_with_drug_ at −0.53 and −0.51 for lungs and −5.2 and −0.43 for spleens (Table 4), respectively. All of the combined therapies were significantly more effective than the single therapy (see Tables S1 and S2). As seen in Tables 2 and 3 and Fig. 2C and G, 99% kill with the RMP-INH-PZA combination was achieved at about 3 weeks for both lungs and spleens, showing elimination rate constants (−0.51 for lung and −0.48 for spleen) similar to those of RMP-INH or RMP-PZA (Table 4). There was no significant difference in efficacies among these RMP-containing regimens against M. tuberculosis in this mouse model (see Tables S1 and S2). All of the RMP-containing combinations achieved undetectable M. tuberculosis CFU counts (Tables 2 and 3) and negative broth growth in selective Kirchner liquid medium in murine lungs and spleens at 14 weeks of treatment. However, when INH was combined with PZA (Fig. 2D and H), there was no noticeably increased initial kill compared to that of each of the single drugs until 4 weeks of treatment, followed by a reduction of CFU count showing a 99% kill at 5.6 weeks posttreatment (Table 2) for lungs and 4 weeks for spleens (Table 3). This was reflected in the estimates for *k*_net_with_drug_ for the INH and PZA combination, which was −0.42 and −0.44 for lungs and spleens, respectively (Table 4). Although the INH and PZA combinations failed to achieve undetectable M. tuberculosis CFU counts in murine lungs after 14 weeks of treatment (Fig. 2D and H), the difference in efficacies between the single-drug treatment and the combination was significant (see Tables S1 and S2).

**TABLE 2:** Bactericidal and sterilizing activities of experimental regimens against M. tuberculosis in mouse lungs

Infection or treatment time point^*a*^	Activity (mean log CFU per lung ± SD) of:							
	Control	RMP	INH	PZA	RMP-INH	RMP-PZA	INH-PZA	RMP-INH-PZA
D0	4.38 ± 0.04							
D14	6.86 ± 0.13							
D21	7.04 ± 0.01							
W2		6.48 ± 0.14	6.83 ± 0.25	6.87 ± 0.13	6.05 ± 0.07	5.66 ± 0.13	6.84 ± 0.04	6.10 ± 0.16
W4		5.40 ± 0.15	5.57 ± 0.37	5.32 ± 0.15	5.05 ± 0.07	4.26 ± 0.08	5.46 ± 0.24	4.63 ± 0.17
W6		5.37 ± 0.29	5.27 ± 0.70	5.19 ± 0.35	3.64 ± 0.12	3.46 ± 0.18	5.16 ± 0.04	3.81 ± 0.14
W8		5.18 ± 0.13	4.89 ± 0.40	5.05 ± 0.15	3.12 ± 0.21	2.73 ± 0.22	3.83 ± 0.07	2.32 ± 0.24
W11					1.20 ± 0.27	0.77 ± 0.48	2.54 ± 0.12	0.63 ± 0.70
W14^*b*^					0	0	1.82 ± 0.42	0

a D0, 2 h postinfection; D14, 14 days postinfection; D21, 21 days postinfection; W2, week 2 posttreatment.

b CFU counts were derived from one-third of the tissue homogenate, and the limit of detection was 3 CFU/lung.

**TABLE 3:** Bactericidal and sterilizing activities of experimental regimens against M. tuberculosis in mouse spleens

Infection or treatment time point^*a*^	Activity (mean log CFU per spleen ± SD) of:							
	Control	RMP	INH	PZA	RMP-INH	RMP-PZA	INH-PZA	RMP-INH-PZA
D0	5.32 ± 0.04							
D14	7.06 ± 0.01							
D21	7.22 ± 0.21							
W2		6.66 ± 0.06	6.85 ± 0.15	6.45 ± 0.51	5.59 ± 0.14	5.07 ± 0.12	6.14 ± 0.17	5.57 ± 0.15
W4		5.49 ± 0.10	5.58 ± 0.30	5.89 ± 0.10	4.52 ± 0.14	3.99 ± 0.22	5.29 ± 0.25	4.15 ± 0.10
W6		4.90 ± 0.24	5.19 ± 0.19	5.46 ± 0.24	3.52 ± 0.20	2.71 ± 0.45	5.01 ± 0.08	3.15 ± 0.29
W8		4.80 ± 0.24	4.99 ± 0.16	5.06 ± 0.08	3.01 ± 0.11	1.95 ± 0.19	4.57 ± 0.06	1.99 ± 0.07
W11					0.78 ± 0.50	0.64 ± 0.69	2.53 ± 0.43	0.73 ± 0.49
W14^*b*^					0	0	1.52 ± 0.50	0

a D0, 2 h postinfection; D14, 14 days postinfection; D21, 21 days postinfection; W2, week 2 posttreatment.

b CFU counts were derived from one-third of the tissue homogenate, and the limit detection was 3 CFU/lung.

**TABLE 4:** Estimates of exponential rate constants during pretreatment and treatment of mouse lungs and spleens

Treatment^*a*^	*k*_net_ value(week^−1^) by sample source^*b*^							
	Lung				Spleen			
	*k*_net_no_drug_		*k*_net_with_drug_		*k*_net_no_drug_		*k*_net_with_drug_	
	Est	%RSE	Est	%RSE	Est	%RSE	Est	%RSE
RMP	1.03	1.99	−0.21	8.22	1.08	3.15	−0.31	6.09
INH	1.03	1.99	−0.27	10.37	1.08	3.15	−0.29	6.35
PZA	1.03	1.99	−0.26	9.05	1.08	3.15	−0.26	5.92
RMP-INH	0.85	5.05	−0.53	2.61	0.58	0.91	−0.52	2.15
RMP-PZA	0.85	5.05	−0.51	1.65	0.58	0.91	−0.43	4.95
INH-PZA	0.85	5.05	−0.42	3.00	0.58	0.91	−0.44	4.38
RMP-INH-PZA	0.85	5.05	−0.51	2.91	0.58	0.91	−0.48	3.23

a Single-drug treatments lasted 8 weeks and double- and triple-drug treatments lasted 14 weeks.

b Est, estimate; %RSE, percent relative standard error.

### Relapse rate of treatment with RMP-INH, RMP-PZA, and RMP-INH-PZA in the modified Cornell mouse model.

After 8 weeks of immunosuppression with high-dosage steroid, disease relapse rates for the treatments with two- and three-drug were determined by the percentage of mice that developed positive M. tuberculosis CFU counts in lungs, spleens, or both. The organ relapse proportions for the four regimens are shown in Table 5. The treatment with the regimens of RMP-INH, RMP-PZA, and RMP-INH-PZA yielded similar relapse rates at 85, 77.3, and 87.5%, respectively. These relapse rates were not significantly different among the three-drug regimens or from a 100% relapse rate (*P* > 0.002 by Fisher's exact test, including Bonferroni correction for multiple pairwise tests). The INH and PZA combination did not produce a negative organ CFU count at the termination of the 14-week treatment (Tables 2 and 3).

**TABLE 5:** Relapse of mice after two- or three-drug treatment

Culture source or parameter measured	Value for^*a*^:		
	RMP-INH	RMP-PZA	RMP-INH-PZA
Positive culture source (no.)			
Spleen only	8	6	15
Lung only	5	4	1
Both	4	7	5
Neither	3	5	3
Total no. of mice with positive cultures	17	17	21
Total no. of mice	20	22	24
Relapse (%)	85	77.3	87.5

a *P* values of relative relapse rates determined by Fisher's exact test were 0.7 (RMP-INH/RMP-PZA), 1.0 (RMP-INH/RMP-INH-PZA), and 0.45 (RMP-PZA/RMP-INH-PZA) with Bonferroni correction. A *P* value of <0.008 was considered significant.

### Determination of persisters after treatment with four drug regimens.

In order to determine the effect of the four combination regimens on the posttreatment level of persisters, we analyzed lung and spleen homogenates at 14 weeks posttreatment using M. tuberculosis culture filtrate resuscitation (6). As shown in Table 6, at 14 weeks posttreatment, although CFU counts and growth in Kirchner liquid medium were negative for the drug regimens INH-RMP, RMP-PZA, and INH-RMP-PZA, there were significant amounts of culture filtrate-dependent persisters present in lungs and spleens (1.89 log cells/lung and 2.09 log cells/spleen for RMP-PZA, 2 log cells/lung and 2.18 log cells/spleen for INH-RMP, and 1.94 log cells/lung and 2.12 log cells/spleen for INH-RMP-PZA). After INH-PZA treatment, there were 4-log culture filtrate-resuscitated bacilli in both lungs and spleens. If we exclude CFU count-positive bacilli, there were still 4-log culture filtrate-dependent persisters in the organs of INH-PZA-treated mice.

**TABLE 6:** Resuscitation of M. tuberculosis H37Rv in mouse lungs and spleens of a modified Cornell mouse model after treatment with different drug regimens

Drug regimen^*a*^	Sample source^*b*^			
	Lung		Spleen	
	Plate count	Broth count RPF	Plate count	Broth count RPF
RMP-PZA	0	1.89 ± 0.12	0	2.09 ± 0.29
INH-RMP	0	2.00 ± 0.14	0	2.18 ± 0.32
INH-RMP-PZA	0	1.94 ± 0.14	0	2.12 ± 0.26
INH-PZA	1.82 ± 0.42	4.10 ± 0.09	1.52 ± 0.5	4.07 ± 0.15

a Fourteen-week treatment.

b Plate counts were determined as CFU counts of the organ homogenies (*n* = 10) on 7H11 agar plates (mean log CFU/organ ± standard deviations). CFU counts were derived from one-third of the tissue homogenate and calculated to represent counts of the entire organ. The limit of detection was 3 CFU/organ. Broth count RPF were determined by MPN of the diluted organ homogenies (*n* = 10) with the culture filtrates (mean log MPN/organ ± standard deviations). Broth counts were derived from one-third of the tissue homogenate and was calculated to represent the MPN of the entire organ. The limit of detection was 10 MPN/organ.

## DISCUSSION

In this study, we reevaluated the current TB treatment regimen and studied the drug interactions by comparing the bacterial elimination rates, the number of culture filtrate-dependent bacteria present at treatment completion, and relapse rates with different therapies in a mouse tuberculosis treatment model based on the model established at Cornell University over a half century ago (8, 9). This model enables us to determine anti-TB activities of combination regimens and, importantly, to measure relapse rates. It is characterized by the inoculation of a large number of bacteria intravenously to initiate an infection and the treatment of the disease once the infection has been established (2 to 3 weeks postinfection). In this model, an intensive treatment is able to render mouse organs culture negative on agar plates and in broth culture lacking culture filtrate, but it fails to prevent relapse (10, 11). However, these apparently culture-negative organs contained viable bacteria that could be cultivated by supplementing broth media with culture filtrate (6) containing RPF (7). Significantly, we found that when RMP was combined with INH, PZA, or INH-PZA, significant additive activities were observed compared to each of the single-drug treatments. However, the combination of INH and PZA showed a less significant additive effect than either of the single-drug treatments. The combination regimens of RMP-INH, RMP-PZA, and RMP-INH-PZA exhibited equivalent treatment efficacies with very similar relapse rates which could not actually be differentiated from a 100% relapse rate, while INH-PZA failed to render organ culture negative after 14 weeks of treatment. Rifampin-containing regimens reduced the number of culture filtrate-dependent persisters to a greater extent than INH-PZA but did not eliminate them from mouse organs by the end of 14 weeks of treatment.

In humans, the key for treatment success depends on the bactericidal drugs INH and RMP, which rapidly kill actively replicating bacilli in cavities and control disease progression (16) within the first 2 months of chemotherapy. This is defined by negative acid-fast staining in sputum. In fact, bactericidal drugs such as INH exhibit bactericidal activity during the first 2 days of monochemotherapy (17). The need for prolonged treatment is due to the emergence of persistent bacilli which may arise in the heterogeneity of host environments (18). These persistent tubercle bacilli are undetectable by the traditional microbiological methods and become profoundly tolerant to bactericidal drugs (10). Sterilizing drugs such as PZA and RMP contribute to shortening of the treatment duration (18). However, in our study comparing elimination rate constants for monotherapies in mice, there was no significant difference between RMP, INH, or PZA. There was no superior bactericidal activity of INH, which contrasts with the effect of INH in humans. This indicates that treatment profiles are different between mice and humans.

Synergistic drug interactions have not been demonstrated in the treatment of TB in mice. It is generally accepted that more than a 2-log kill compared to the single drug defines a synergistic combination (19). Here, we showed that enhanced bactericidal activities were achieved when RMP was combined with INH or PZA. Estimates of the elimination rate constant for all the combinations were significantly faster (*P* < 0.0001) than those of all single drugs (see Tables S1 and S2 in the supplemental material), showing a 99% kill of the bacilli (a 2-log kill) achieved 4 to 5 weeks earlier than that with monotherapies. The activities of the combinations, namely, RMP-INH, RMP-PZA, and RMP-INH-PZA, shown by the value of the exponential elimination rate constant (Table 4) demonstrated significant additive interactions on the original scale. Therefore, it is interesting that the INH-PZA combination showed less enhanced effect than the singly dosed drugs at the earlier stage of treatment when there was a large number of actively growing organisms (10), and its increased efficacy compared to the monotherapies was more apparent after 6 weeks of treatment. This was in agreement with the previous findings that the INH and PZA combination was more efficacious than the single drug in the reduction of organ bacterial counts and prevention of relapse rates in mice (8, 20) and in humans (21–23). The efficacies of all RMP-containing regimens (INH-RMP, RMP-PZA, and INH-RMP-PZA) in mouse tuberculosis treatment were very similar (*P* > 0.05), as shown by the similarity of the elimination rate constants, which confirmed previous findings (3, 4), while INH-PZA therapy was less effective than other combination therapies (*P* < 0.001) (5). At the end of 14 weeks of treatment, lungs and spleens of mice treated with RMP-INH, RMP-PZA, or RMP-INH-PZA became CFU count and broth count negative; conversely, the INH and PZA combination failed to achieve culture negativity in the mouse organs. After 8 weeks of steroid treatment, tubercle bacilli were found in the organs of mice treated with RMP-INH, RMP-PZA, or RMP-INH-PZA. Although the elimination rates of the rifampin-containing regimens (RMP-INH, RMP-PZA, and RMP-INH-PZA) displayed significant differences from that for INH-PZA (the latter regimen having failed to achieve culture negativity), their relapse rates could not be differentiated from a 100% relapse rate at the numbers of mice examined in this study. This is attributable to the presence of persistent bacteria in the RMP-containing regimens which could only be resuscitated by culture filtrate (Table 6). This observation coincided with the previous finding that early bactericidal activities of certain novel drug regimens were not necessarily predictive of a sterilizing effect (24), which may be attributed to the inability of the drug regimens to eliminate the persistent bacilli which were undetectable using our traditional microbiological methods. Recently, we showed that faster elimination rates derived from high-dose RMP treatment led to the elimination of persistent bacteria, and this contributed to a shortened chemotherapy and a reduced relapse rate (6). It is not known if the elimination rate of culture filtrate-dependent bacteria is a likely surrogate measure of the sterilizing activity of the regimens, as this has not been determined. RMP-containing regimens resulted in faster elimination rates than INH-PZA against plate-cultivable and reduced culture filtrate-dependent subpopulations at 14 weeks of treatment. Clearly further study is required to demonstrate if the elimination rate of culture filtrate-dependent bacteria is a better surrogate for sterilizing effect.

The major caveat of this study was the relatively short period of chemotherapy in which INH-PZA failed to achieve CFU count-negative mouse organs. This made it difficult to compare relapse rates of all the treatment regimens. It is likely that a difference in the sterilizing activity of these regimens would emerge with longer durations of treatment. Future work aiming to use a larger number of mice and longer treatment duration would illustrate more clearly the relationship between elimination rate and relapse among different drug regimens.

Bacterial population dynamics in infected animals is expected to be complex and related to the density and composition of the infecting population. In this study, the route of infection was systemic, and it was performed according to a previously established method (8, 9). Previous studies showed that intravenous infection of M. tuberculosis in mice led to slower disease progression in lungs (25) in spite of a high level of systemic immunity. However, low-dose aerosol infection resulted in substantially greater virulence of M. tuberculosis in mouse lungs (25). In aerosol-infected mice, small numbers of bacilli were seeded in the lung, and these then multiply into larger populations (25), presumably with smaller subpopulations of persistent organisms. It has been shown that slower bactericidal rates of combination regimens were found in intravenously infected mice with a higher relapse rate than aerosol-infected animals (26). The difference might be due to different immune responses produced between intravenously and aerosol-infected animals. It is not known if different routes of infection affect the level of culture filtrate-dependent persisters. Future work will be conducted to compare persistent M. tuberculosis levels in mice using respiratory and systemic infections.

It has been shown that antagonism occurred between INH and the RMP-PZA combination in the treatment of tuberculosis in mice (4). The authors suggested that the antagonistic effect was partially derived from the interaction of INH with RMP, as the addition of INH significantly reduced the *C*_max_ and AUC of RMP (4). There was also a negative interaction between INH and PZA against M. tuberculosis (5) in mice when a higher dose of INH was used. In contrast, a separate study showed that RMP-PZA was less effective than the RMP-INH-PZA combination in mouse models with both aerosol and intravenous infections, indicating that the inclusion of INH in the regimen showed no negative interaction with RMP-PZA (26). In observations of CFU counts over time with RMP-INH, RMP-PZA, and RMP-INH-PZA, RMP-PZA treatment showed increased reduction in CFU counts compared to RMP-INH and RMP-INH-PZA, especially at weeks 2, 4, and 6 of treatment (Fig. 2), indicating that INH was slightly antagonistic. However, our data demonstrated that this antagonistic effect when INH is added to the RMP-PZA regimen was not significant based on a comparison of the elimination rate constants estimated from the profiles of bacterial elimination over time; the *k*_net_with_drug_ was −0.51 for RMP-PZA and −0.51 for RMP-INH-PZA (a *P* value of >0.002 indicates significant difference). We also observed that the INH-PZA combination was not antagonistic against M. tuberculosis compared to the activities of each single drug. The differences in drug interaction of the current regimens seen from different studies may be attributable to different experimental conditions, such as the use of different M. tuberculosis strains, mouse species, routes of infection, and length of treatment used by different research groups (26). Importantly, our demonstration of RMP-containing regimens being superior to an RMP-free regimen against M. tuberculosis in the modified Cornell mouse model indicated the essential role RMP plays in the current regimen to treat tuberculosis disease. However, the relationship between elimination rate, MPN counts, and relapse rates requires further evaluation across a broader range of (possibly non-RMP-containing) regimens.

## Supplementary Material

Supplemental material
