# Supplementary material for: Investigation of Elimination Rate, Persistent Subpopulation Removal, and Relapse Rates of Mycobacterium tuberculosis by Using Combinations of First-Line Drugs in a Modified Cornell Mouse Model
Source: Antimicrob Agents Chemother. 2016 Jul 22;60(8):4778–85. doi: 10.1128/AAC.02548-15 (PMC4958161; doi:10.1128/AAC.02548-15)
Supplement: Supplemental material [file supp_60_8_4778__index.html]

Investigation of Elimination Rate, Persistent Subpopulation Removal, and Relapse Rates of Mycobacterium tuberculosis by Using Combinations of First-Line Drugs in a Modified Cornell Mouse Model — Supplemental material 

# Investigation of Elimination Rate, Persistent Subpopulation Removal, and Relapse Rates of Mycobacterium tuberculosis by Using Combinations of First-Line Drugs in a Modified Cornell Mouse Model

## Supplemental material

- Supplemental file 1 -

  Tables S1 and S2

  PDF, 30K
