# Supplementary material for: Investigation of Elimination Rate, Persistent Subpopulation Removal, and Relapse Rates of Mycobacterium tuberculosis by Using Combinations of First-Line Drugs in a Modified Cornell Mouse Model
Source: Antimicrob Agents Chemother. 2016 Jul 22;60(8):4778–85. doi: 10.1128/AAC.02548-15 (PMC4958161; doi:10.1128/AAC.02548-15)
Supplement: Supplemental material [file AAC.02548-15_zac008165395so1.pdf]

Table S1. Pairwise comparisons of differences between elimination rate constants during therapy in mouse lungs

Treatment	diff. knet_with_drug			
	est.	se.	p-value	
RMP vs. RMP-INH	0.32	0.02	P < 0.0001	significant difference
RMP vs. RMP-PZA	0.3	0.02	P < 0.0001	significant difference
RMP vs. INH-PZA	0.2	0.02	P < 0.0001	significant difference
RMP vs. RMP-INH-PZA	0.29	0.02	P < 0.0001	significant difference
INH vs. RMP-INH	0.26	0.03	P < 0.0001	significant difference
INH vs. INH-PZA	0.15	0.03	P < 0.0001	significant difference
INH vs. RMP-PZA	0.25	0.03	P < 0.0001	significant difference
INH vs. RMP-INH-PZA	0.24	0.03	P < 0.0001	significant difference
PZA vs. RMP-PZA	0.26	0.02	P < 0.0001	significant difference
PZA vs. INH-PZA	0.16	0.03	P < 0.0001	significant difference
PZA vs. RMP-INH	0.28	0.03	P < 0.0001	significant difference
PZA vs. RMP-INH-PZA	0.25	0.03	P < 0.0001	significant difference
RMP vs. INH	0.06	0.03	P > 0.002	no significant difference
RMP vs. PZA	0.04	0.03	P > 0.002	no significant difference
PZA vs. INH	0.01	0.04	P > 0.002	no significant difference
RMP-INH vs. RMP-PZA	0.02	0.02	P > 0.002	no significant difference
RMP-INH vs. INH-PZA	0.12	0.02	P < 0.0001	significant difference
RMP-INH vs. RMP-INH-PZA	0.02	0.02	P > 0.002	no significant difference
RMP-PZA vs. INH-PZA	0.1	0.02	P < 0.0001	significant difference
RMP-PZA vs. RMP-INH-PZA	0.01	0.02	P > 0.002	no significant difference
INH-PZA vs. RMP-INH-PZA	0.09	0.02	P < 0.0001	significant difference

Table S2. Pairwise comparisons of differences between spleen elimination rate constants during therapy in muse spleens

Treatment	diff. knet_with_drug			
	est.	se.	p-value	
RMP vs. RMP-INH	0.21	0.02	P < 0.0001	significant difference
RMP vs. RMP-PZA	0.12	0.03	P < 0.0001	significant difference
RMP vs. INH-PZA	0.13	0.03	P < 0.0001	significant difference
RMP vs. RMP-INH-PZA	0.17	0.02	P < 0.0001	significant difference
INH vs. RMP-INH	0.23	0.02	P < 0.0001	significant difference
INH vs. INH-PZA	0.15	0.03	P < 0.0001	significant difference
INH vs. RMP-PZA	0.14	0.03	P < 0.0001	significant difference
INH vs. RMP-INH-PZA	0.19	0.02	P < 0.0001	significant difference
PZA vs. RMP-PZA	0.17	0.03	P < 0.0001	significant difference
PZA vs. INH-PZA	0.18	0.02	P < 0.0001	significant difference
PZA vs. RMP-INH	0.26	0.02	P < 0.0001	significant difference
PZA vs. RMP-INH-PZA	0.22	0.02	P < 0.0001	significant difference
RMP vs. INH	-0.02	0.03	P > 0.05	no significant difference
RMP vs. PZA	-0.05	0.02	P > 0.05	no significant difference
PZA vs. INH	0.03	0.02	P > 0.05	no significant difference
RMP-INH vs. RMP-PZA	-0.09	0.02	P > 0.04	no significant difference

RMP-INH vs. INH-PZA	-0.08	0.02	P > 0.04	no significant difference
RMP-INH vs. RMP-INH-PZA	-0.04	0.02	P > 0.05	no significant difference
RMP-PZA vs. INH-PZA	-0.01	0.03	P > 0.05	no significant difference
RMP-PZA vs. RMP-INH-PZA	-0.05	0.03	P > 0.05	no significant difference
INH-PZA vs. RMP-INH-PZA	0.04	0.02	P > 0.05	no significant difference
